# Genomic analysis of emerging pathogens: methods, application and future trends

**DOI:** 10.1186/s13059-014-0541-9

**Published:** 2014-11-22

**Authors:** Lucy M Li, Nicholas C Grassly, Christophe Fraser

**Affiliations:** Department of Infectious Disease Epidemiology, Imperial College London, St Mary’s Campus, London, W2 1PG UK

## Abstract

The number of emerging infectious diseases is increasing. Characterizing novel or re-emerging infections is aided by the availability of pathogen genomes. In this review, we evaluate methods that exploit pathogen sequences and the contribution of genomic analysis to understand the epidemiology of recently emerged infectious diseases.

## Introduction

When a pathogen crosses over from animals to humans, or an existing human disease suddenly increases in incidence, the infectious disease is said to be ‘emerging’. The number of emerging infectious diseases (EIDs) has increased over the last few decades, driven by both anthropogenic and environmental factors [1]. These include the expansion of agricultural land, which increases the exposure of livestock and humans to infections in wildlife [2]; a greater volume of air traffic, enabling EIDs to rapidly spread across the world [3,4]; and climate change, which alters the ecology and density of animal vectors, thereby introducing diseases to new geographic locations [5]. Novel strains of existing pathogens also have the potential to cause large epidemics. The over- and misuse of antimicrobial drugs have contributed to the growing number of drug-resistant pathogen strains [6,7].

Detecting, characterizing and responding to an EID requires co-ordination and collaboration between multiple sectors and disciplines. Laboratory-based research helps to characterize the pathogen and its interactions with host cells, but is less useful for quantitative understanding of population-level disease dynamics. Modeling approaches enable a large number of hypotheses to be tested, which might not be logistically or ethically feasible in laboratory and field experiments. In addition to characterizing past disease dynamics, modeling future trends informs decisions regarding outbreak response and resource allocation [8]. Modeling plays an especially important role in epidemiological studies of infectious disease spread, because the transmission of infectious disease between individuals is not directly observable. At the individual level, transmission times and who infected whom are typically unknown. And at the population level, disease burden needs to be inferred from observable data. Important public health questions such as how quickly an epidemic spreads and how many people will be infected are hard to quantify without a mechanistic understanding of underlying factors driving disease transmission. By expressing disease spread in mathematical terms, statistical properties of epidemics can be estimated to help address specific questions regarding disease spread and control efforts [9].

Another discipline contributing to the study of EIDs is pathogen genomics. As sequencing technology has become more accessible and affordable, genetic analysis has played an increasingly important role in infectious disease research. Sequencing pathogens can confirm suspected cases of an infectious disease, discriminate between different strains, and classify novel pathogens. In addition to examining individual pathogen sequences, multiple sequences can be analyzed together using phylogenetic methods to elucidate evolutionary [10] and transmission [11] history. Just as mathematical models of disease transmission help to capture the epidemiological properties of an infectious disease, modeling the molecular evolution of pathogen genomes is important for phylogenetic methods.

Besides characterizing the genetics and evolution of a pathogen, mathematical models used in population genetics link demographic and evolutionary processes to temporal changes in population-level genetic diversity. The coalescent population genetics framework was developed so that demographic history could be inferred from the shape of the genealogy linking sampled individuals [12,13]. More recently, the birth-death model has been applied to infectious diseases to infer epidemiological history from a genealogy [14,15]. Given the link between pathogen evolution and disease transmission, there is a trend towards integrating both epidemiologic and genetic data in the same analytical framework [16-18].

In this review, we provide an overview of recent developments in genomic methods in the context of infectious diseases, evaluate integrative methods that incorporate genetic data in epidemiological analysis, and discuss the application of these methods to EIDs.

## Role of genetics in studying infectious diseases

**Figure 1:**
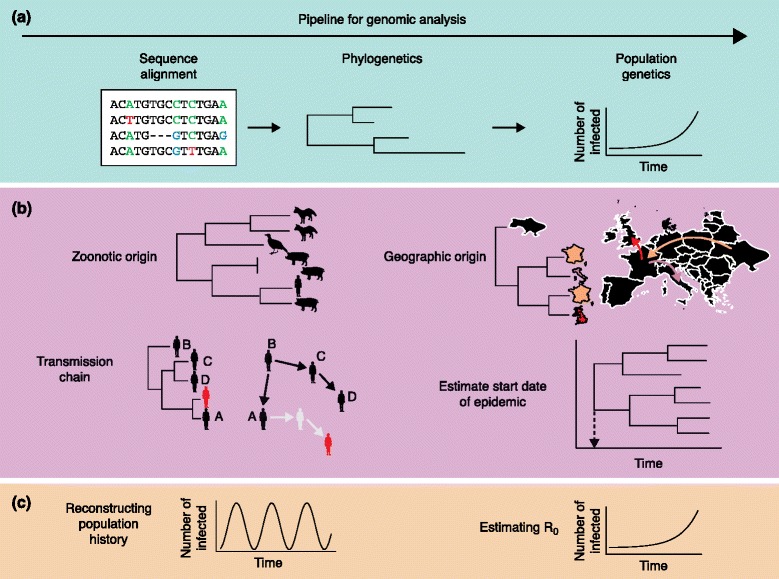
**Contribution of genomic analysis to epidemiological studies of emerging infectious diseases. (a)** Genomic analysis begins with obtaining a multiple sequence alignment of pathogen sequences from which a phylogeny can be built to represent the evolutionary relationship between samples. Further population genetic analysis using the coalescent framework can reveal the population history of the pathogen based on the sample phylogeny. **(b)** Coupling phylogeny with additional information is useful for uncovering zoonotic origins, the spatiotemporal patterns of disease spread, and transmission chains. The results of such phylogenetic analysis should be interpreted with care as the direction of transmission is not always clear and there might exist missing intermediate links. **(c)** Coalescent analysis of pathogen genealogy is used to characterize past epidemiological dynamics and estimate epidemiological parameters, such as the reproductive number.

Over the last two decades, sequence data have increased in quality, length and volume due to improvements in the underlying technology and decreasing costs. As a result, pathogen sequences are regularly collected during routine surveillance and clinical studies. Just as mathematical modeling can be used to analyze surveillance data to reveal details of disease transmission (Box 1), analysis of pathogen genomes employs mathematical frameworks to elucidate pathogen biology, evolution and ecology (Figure 1).

At the most basic level, mathematical models are used to find the optimal alignment of pathogen sequences. Multiple sequence alignment is useful for finding highly conserved or variable regions, shedding light on the molecular biology of the pathogen. Furthermore, coupling sequences with clinical information can help identify the contribution of polymorphic sites to disease. Revealing the evolutionary history of a pathogen requires a quantitative description of relatedness. Based on polymorphic sites in the sequence alignment, a model of sequence evolution is then used to reconstruct the phylogeny [19]. Often, there is insufficient genetic diversity in the sample to fully infer the phylogeny without ambiguity. In such a case, it is useful to consider a tree as an unknown set of parameters and obtain its posterior probability distribution using a Bayesian framework, such as the Markov Chain Monte Carlo (MCMC) approaches [20,21].

Biological samples from which pathogen genetic material is sequenced are usually associated with geographic or temporal information (Figure 1b). When this additional information is available, phylogenetic methods can reveal the spatiotemporal spread of the pathogen in the population. If an outbreak is densely sampled, then the pathogen phylogeny provides information about the underlying transmission network and helps to uncover who infected whom [22,23], though phylogenetic clustering alone is usually not sufficient to prove direct transmission or direction of infection (Figure 1b).

Incorporating sampling times helps to convert a phylogeny specified in units of nucleotide substitutions to a phylogeny specified in units of time [24]. The conversion is straightforward if sequence evolution follows a strict molecular clock, whereby the rate of substitution remains constant over time. However, selection pressure and population bottlenecks can lead to changes in the rate of substitution [25]. More flexible models have been developed to incorporate time-varying rates of evolution [26,27]. With branch lengths in units of real time, the start date of an epidemic can be estimated. Whereas phylogenetics aims to delineate the relationship between individuals, population genetics aims to link population processes to observed patterns of genetic diversity. Inferences regarding pathogen population history are based on the genealogy, or ancestry, of sequences from sampled individuals, and often carried out in a retrospective population genetics framework known as the coalescent [12] (Box 2). A genealogy describes the ancestry of sampled individuals. Going backwards in time, pairs of lineages coalesce when they share a common ancestor, until the last two lineages coalesce at the time of the most recent common ancestor (TMRCA) for the entire sample.

Since the turn of the century, the coalescent has been increasingly applied to infectious disease research to infer epidemic history from pathogen sequences, thereby linking pathogen evolutionary history to disease epidemiology (Figure 1c). The method is especially useful for analyzing infectious diseases with mild or asymptomatic infections, for which case-based surveillance data severely underestimate prevalence, because the coalescent assumes a small sample compared to the population size [28-30].

Other approaches have been developed to make epidemiological inferences from genetic data. Of particular note is the birth-death model [31], which describes the rates of transmissions, recoveries and deaths, and sampling events in terms of the sample genealogy [14]. Just as there are coalescent methods incorporating population structure [32-34] and compartmental models [35-37], similar methods exist in the birth-death framework [38,39]. Unlike the coalescent framework, the birth-death model is still valid for densely sampled populations, which makes it more useful for studying small outbreaks. However, accurately inferring epidemiological parameters depends on correctly specified sampling proportions [40]. Although the two approaches are methodologically different, both aim to reconstruct pathogen population history and produce estimates of epidemiological parameters, such as the reproductive number (R_0_). The focus on the coalescent framework in this review is due to its more pervasive use in the literature and its greater versatility when integrated with epidemiological models compared to birth-death models.

Because of the simplistic assumptions of population genetics models, the population size inferred using coalescent-based methods cannot be directly interpreted as pathogen population size (prevalence of infection). It is rather the effective population size, N_e_ (Box 2), which refers to the size of a Wright-Fisher population that would produce the same level of genetic diversity as observed in the sample. In real populations, the variance of the offspring distribution (Box 1) is higher than expected in a Wright-Fisher population due to heterogeneity in host infectiousness, non-random mixing of the population, and migration events. The consequence of a large variance is that there is a greater discrepancy between the effective and census population sizes [41]. Accounting for the dispersion of the offspring distribution is especially important when analyzing infectious disease data because of the widespread occurrence of transmission heterogeneity [42].

Another statistical property of epidemics affecting the results of modeling studies is the generation time distribution, which describes the time between infection of the primary case and of secondary cases. Obtaining an estimate of the generation time is important for two reasons. First, estimates of R_0_ from the initial growth rate of an epidemic depend on the generation time distribution [43]. As R_0_ is the mean of the offspring distribution, its value affects the relationship between the effective population size, N_e_, and the census population size, N. Second, the coalescent model was originally specified in units of generations, and so estimates in this framework need to be converted to natural units using the generation time, T_g_.

Because transmission events are rarely observed, the generation time distribution is often approximated by the distribution of the serial interval, which is the time between onset of symptoms in the primary and secondary cases. The two distributions generally share the same mean but might have different variances [44]. Furthermore, the observed generation time decreases as the epidemic grows but increases again after the epidemic peak due to right censoring [45].

## Integrating genetics with other data

As both sequence and surveillance data contain information regarding the transmission process, simultaneously analyzing both datasets should yield more accurate estimates of epidemiological parameters than separate analyses [17]. The recently established discipline of phylodynamics takes an interdisciplinary approach to understand the pathogen phylogenetics and epidemiology in terms of disease transmission.

Most efforts thus far have focused on enhancing phylogenetic and population genetic analyses by incorporating spatial and temporal information about the sequences. The molecular clock model assumes a constant rate of evolution and thus helps to estimate the time of the most recent common ancestor of the sample, which approximates the start date of an epidemic. Molecular clock analysis has been used to date the emergence of a range of emerging pathogens from HIV [46] to multidrug-resistant *Streptococcus pneumoniae* [47].

Linking geographic information with sequences can reveal the spatial spread of infectious disease. Phylogenetic reconstruction of seasonal influenza (H3N2) sequences has revealed the contribution of viral circulation in temperate regions to the global genetic diversity of influenza, and determined that not all epidemics in temperate regions are seeded by strains from South East Asia [48,49]. Also using global sequences, hepatitis C virus (HCV) subtypes were shown to spread from developed to developing countries [50]. Finally, phylogeographic analysis of methicillin-resistant *Staphylococcus aureus* samples identified England as the source of the EMRSA-15 lineage [51].

By contrast, there have been relatively few studies incorporating genetic data into epidemiological frameworks. Although genetic analysis plays an important role in elucidating transmission links in disease outbreaks [20,21,52], its integration with epidemiological models to understand population-level disease dynamics has been more limited. In one of the first papers to link coalescent inference to mathematical models in epidemiology, the effective population sizes of HIV-1 subtypes A and B were estimated from the maximum likelihood trees of viral sequences [53]. In addition to revealing population sizes, Pybus *et al*. [54] estimated the R_0_ values of HCV subtypes (1a, 1b, 4 and 6) by inferring the epidemic growth rate from viral genealogy. Taking integration a step further, the coalescent process has been described for compartmental epidemiological models such as the Susceptible-Infected-Recovered (SIR) model, thereby enabling epidemiological parameters to be inferred from the genealogy [35]. To infer demographic history from both pathogen genomes and epidemiological data, Rasmussen *et al*. [17] developed a Markovian framework in which the population size at each time step was estimated by taking into account both the surveillance data and the genealogy. The epidemic history reconstructed using both datasets was more accurate than when analyzing each type of data separately.

In all the above methods, the genealogy of the sampled sequences was fixed. However, there might be great uncertainty regarding the order and the timing of coalescence, especially if the sequences are sampled within a short time period. While genealogical reconstruction using Bayesian MCMC approaches allows phylogenetic uncertainty to be incorporated into estimates of population size [13,31], an integrative model is lacking in which uncertainties arising from both genetic and epidemiological data are incorporated during demographic reconstruction.

## Application to emerging pathogens

Models of pathogen evolution and mechanistic models of disease spread have increased in complexity. There is also greater computational power to test these models with data. However, these sophisticated models have mostly been applied to infectious diseases for which abundant data are available. For example, new methods are most often tested on the HIV-1 pandemic [15,34,35,55], for which data have been extensively collected from various settings and sources since the virus was first characterized three decades ago. It is worthwhile to evaluate how genomic methods have been applied to other diseases that have emerged more recently. In this section, we will present three case studies of recently emerged infectious diseases to illustrate the power and shortcomings of genomic methods discussed in this review.

### Ebola virus emergence in West Africa

Since emerging in Guinea in March 2014, Ebola virus (EBOV) has spread to other countries in Western Africa, resulting in the largest outbreak of Ebola since it was first identified in 1976. The first viral genomes were made available just a month after alarm was raised about a new Ebola outbreak in Guinea [56], with further sequences collected in Sierra Leone [57]. By aligning all the genomes, a number of polymorphic sites were identified, including eight in highly conserved regions of the genome. Further association studies are needed to clarify the role of these genetic variants in determining disease outcome. Using the sampling dates of the sequences and a molecular clock model, phylogenetic analysis of 81 EBOV sequences revealed a start date of February 2014 in Guinea, spreading to Sierra Leone by April 2014 [57].

Uncovering the relationship between the 2014 EBOV lineage and previous EBOV outbreaks has proved trickier than understanding the disease dynamics during the 2014 outbreak. Initial phylogenetic analysis suggested that lineages causing the present outbreak did not cluster with EBOV strains that caused earlier outbreaks in Central Africa [56]. However, Dudas and Rambaut [58] noted that the divergence of Guinea sequences from those of previous outbreaks was because they were sequenced most recently and had accumulated the highest number of substitutions. Assuming that the EBOV genome followed a molecular clock model, the authors re-rooted the tree to a lineage that caused an outbreak in 1976 [58]. Instead of silently circulating in West Africa, the EBOV lineage causing the current outbreak likely descended from a lineage that previously caused outbreaks in the Democratic Republic of Congo.

These studies highlight two issues. First, correct rooting of a phylogeny is important for accurate inference of past epidemic history. Correct rooting can be achieved by using an out-group, but one was not available in the case of this EBOV strain. This leads onto the second issue. Without sequences from animal hosts, the mechanism by which EBOV was sustained between outbreaks remains unknown.

### Middle East respiratory syndrome coronavirus

Middle East respiratory syndrome coronavirus (MERS-CoV) first appeared in Saudi Arabia in 2012, and has since been reported in several neighboring countries in the Arabian Peninsula and on other continents [59].

Despite the dearth of sequence data, coalescent-based analysis of 10 genomic sequences produced estimates of the TMRCA (March 2012; 95% confidence interval (CI): November 2011 to June 2012), R_0_ (1.21; 95% CI: 1.08, 1.40), and doubling time (43 days; 95% CI: 23, 104 days) [60]. Without further sequencing of the animal reservoirs, the authors could not infer whether these estimates applied to the animal reservoir or the human epidemic, because the methods are agnostic as to where transmission and evolution occur. The credible intervals around the estimates were unsurprisingly large given the small sample size.

Unlike the 2014 EBOV outbreak, which is sustained by human-to-human transmission [57], there appears to have been multiple introductions of MERS-CoV into the human population. Identification of the animal reservoir is therefore crucial for establishing risk factors of infection and planning appropriate interventions to control the disease. Since bats are reservoirs for other coronaviruses, their being a reservoir host is possible. A 182-nucleotide-long region of the RNA-dependent RNA polymerase gene was found to be 100% identical between a viral sample from a patient in Saudi Arabia and from a bat nearby, though the region is known to be highly conserved [61]. However, antibodies against human MERS-CoV have been detected in dromedary camels [62], the camel MERS-CoV genome is similar to human MERS-CoV [62], and there are reports of close contact between patients and camels [63]. Phylogenetic analysis of coronavirus sequences from bats, dromedaries and humans indicate a bat origin, with dromedary camel as an intermediate host [64]. It is possible that there are other animal reservoirs not yet sampled, which highlights the need to carry out extensive animal surveillance to characterize the emergence of an infection in humans.

### Unraveling the complex evolutionary history of pandemic H1N1 influenza

With sequences collected over three decades from humans, pigs and birds, the origin of the pandemic H1N1 influenza A strain (pdmH1N1 or ‘swine flu’) was elucidated soon after emergence. Within two months of the first reported case of swine flu in humans, genomic analysis of the novel influenza strain had been carried out. A phylogeny was constructed for each of the eight genomic segments with sequences from humans, swine and birds. Comparison of these eight phylogenies revealed a complex history of reassortment with a mixture of gene segments from all three groups. The start of the pandemic was estimated to be the end of 2008 or early 2009, and the dates of the reassortment events leading to pdmH1N1 were also obtained [10]. Without good surveillance of influenza in the animal reservoir, the origin of the novel strain would have been difficult to uncover.

By analyzing 11 hemagglutinin sequences collected over a one-month period, the start date of the epidemic was estimated to be in late January 2009 [65]. Repeating the phylogenetic and molecular clock analyses with a further 12 sequences shifted the estimated start date two weeks earlier. Fitting an exponential growth model to the sequence data, R_0_ was estimated to be 1.22, slightly lower than inferred from epidemiological data but with overlapping confidence intervals.

To determine at which point during the pandemic coalescent analysis would have provided accurate and precise estimates of evolutionary rate, R_0_ and TMRCA, real-time estimates of these parameters were obtained for genomic sequences collected in North America [66]. Accurate estimates could have been obtained as early as May, when 100 viral genomes had been sequenced. More precise estimates could have been obtained by the end of June, when 164 had been sequenced. However, inclusion of more sequences of longer length only slightly improved the accuracy of initial estimates [66].

## Future directions

Most statistical models in population genetics have focused on the application of such methods to viruses, although this bias is perhaps unsurprising given the large proportion of EIDs caused by viruses [1]. Whole-genome sequencing of bacterial isolates is becoming more widespread, and can help to uncover genetic determinants of clinical severity, elucidate pathogen-host interactions, and quantify evolutionary rates at within- and between-host levels [67]. Epidemiological investigations using bacterial genomes have also been possible. Even though bacteria acquire point mutations at a lower rate per base than viruses, longer bacterial genomes have provided sufficient genetic resolution for phylogenetic analysis. For example, whole-genome sequencing has been used to refine the tuberculosis transmission network built using contact information [21], and to investigate an outbreak of methicillin-resistant *Staphylococcus aureus* in a hospital and surrounding community in near real-time [68]. The need for longer sequences when conducting epidemiological studies of bacterial infections adds to the per-sample cost of sequencing, and more computational resources are required for coalescent-based inference of pathogen history. However, this latter limitation may be overcome by only analyzing polymorphic sites if samples are similar.

Demographic reconstruction of emerging bacterial pathogens using coalescent-based approaches has been limited compared to work on viral pathogens. In one such study, the temporal changes in genetic diversity of *Streptococcus pneumoniae* in Iceland were estimated based on the coalescent model [47]. This study was limited to a single multidrug-resistant lineage in a single location, with data collected over decades. Over longer evolutionary time-scales, the accumulation of diversity through recombination can obscure phylogenetic relationships. More complex evolutionary models would be required to taken into account these genomic changes, increasing the uncertainty surrounding demographic estimates from genomic data.

In addition to performing analyses with longer sequences, there is also a need to develop methods that exploit as many sequences in the sample as possible. For population studies, available sequences are often subsampled to remove individuals from the same household or in the same close contact network to have a representative sample of the population. Furthermore, sequences from the same individuals are often discarded, though these may be informative for within-host evolution. Although some effort has been made to link within-host to between-host evolution [52,69], the effect of within-host evolution on population genetic inference is still not well studied. Combining analyses across different scales could improve the accuracy of epidemiological predictions and provide better mechanistic explanations of observed trends.

## Conclusion

Genomic studies have contributed to better understanding of EIDs and their spatiotemporal spread. Sophisticated statistical methods have been developed to uncover the epidemiological features of infectious diseases based on the genealogy of their sequences. There is also growing effort to integrate genomic analysis with analysis of epidemiological data. In recent cases of EIDs, genomic data have helped to classify and characterize the pathogen, uncover the population history of the disease, and produce estimates of epidemiological parameters.

## Box 1. Key concepts in mathematical modeling of infectious disease transmission

Representing infectious disease transmission in a mathematical framework requires distilling complex observations into simple but informative expressions. Perhaps the most important statistical property of interest to an epidemiologist is the basic reproductive number, R_0_, which represents the mean number of secondary infections caused by each infected individual in a wholly susceptible population. An epidemic can only occur if R_0_ > 1. As an epidemic progresses, or if there is pre-existing immunity in a population, R_0_ is no longer appropriate for describing the number of secondary infections per primary infection. Instead the effective reproductive number, R, is used. Another important statistical property of an epidemic is the generation time, T_g_, which is the mean time between when an individual becomes infected and when they infect others. The combination of R_0_ and T_g_ provides an indication of how quickly an epidemic will spread.

The most common type of model used in infectious disease research is the compartmental model. Given a set of parameters, a compartmental model tracks the temporal dynamics of subpopulations that are characterized by disease status. For example, a Susceptible-Infected-Recovered (SIR) model describes the changes in the number of susceptible, infected and recovered (and immune) individuals. R_0_ can be calculated by inferring the set of model parameters that can generate the epidemiological dynamics most similar to those observed in the data.

Increasingly, model parameters are inferred in a Bayesian framework. Bayesian inference finds the posterior probability distribution of parameters, given prior information and the data. Exploring all possible parameter combinations is intractable. The use of Markov Chain Monte Carlo (MCMC) for Bayesian statistical inference has enabled efficient estimation of the posterior probability distribution when the distribution cannot be computed analytically [70].

Obtaining estimates of R_0_ and T_g_ is not always sufficient to predict epidemic trajectory if there is significant heterogeneity between individuals. The offspring distribution with mean R and variance σ^2^ describes the probability distribution of the number of secondary infections caused by each infected individual. In compartmental models, the offspring distribution is not explicitly specified but follows from the specification of the model - in the case of the SIR model it follows a geometric distribution. For certain diseases, the offspring distribution is more dispersed than captured by the geometric distribution [42]. In other words, most individuals cause no further infections whereas a few individuals are super-spreaders who cause the majority of infections. Accurate estimate of σ^2^ is important for predicting epidemic outcome and assessing control measures.

## Box 2. Coalescent inference from genetic data

Just as compartmental models can be fitted to surveillance data to infer the epidemiological dynamics of an infectious disease (Box 1), the coalescent framework allows inference of population history from pathogen sequences. The coalescent model describes the statistical properties of the genealogy underlying a small sample of individuals from a large population. In the simplest case, the forward-time dynamics of the population is assumed to follow the Wright-Fisher model, in which the haploid population has discrete, non-overlapping generations, undergoes neutral evolution, and remains the same size [71,72]. Extensions to the coalescent have assumed more complex population dynamics described by deterministic population equations [73], compartmental disease models [35], or non-parametric approaches [13,55,74,75].

Within this framework, going backwards in time, individuals in the current generation are randomly assigned to parents in the previous generation. If two individuals have the same parent, then a coalescent event has occurred. Eventually, all lineages in the sample coalesce to a single individual known as the most recent common ancestor of the sample.

The rate of coalescence is inversely related to population size. If the population follows the Wright-Fisher model, evolutionary changes are selectively neutral, so the shape of the genealogy reflects only demographic changes.
